# A neuronal imaging dataset for deep learning in the reconstruction of single-neuron axons

**DOI:** 10.3389/fninf.2025.1628030

**Published:** 2025-08-05

**Authors:** Liya Li, Ying Hu, Xiaojun Wang, Pei Sun, Tingwei Quan

**Affiliations:** ^1^School of Mathematics and Statistics, Hubei University of Education, Wuhan, Hubei, China; ^2^Institute of Big Data Analysis and Applied Mathematics, Hubei University of Education, Wuhan, Hubei, China; ^3^Key Laboratory of Biomedical Engineering of Hainan Province, School of Biomedical Engineering, Hainan University, Sanya, China; ^4^Department of Clinical Research Institute, Central People’s Hospital of Zhanjiang, Zhanjiang, Guangdong, China; ^5^Britton Chance Center for Biomedical Photonics, Wuhan National Laboratory for Optoelectronics-Huazhong University of Science and Technology, Wuhan, Hubei, China

**Keywords:** neuron morphology, axon reconstruction, deep learning, neuronal imaging datasets, segmentation network

## Abstract

Neuron reconstruction is a critical step in quantifying neuronal structures from imaging data. Advances in molecular labeling techniques and optical imaging technologies have spurred extensive research into the patterns of long-range neuronal projections. However, mapping these projections incurs significant costs, as large-scale reconstruction of individual axonal arbors remains time-consuming. In this study, we present a dataset comprising axon imaging volumes along with corresponding annotations to facilitate the evaluation and development of axon reconstruction algorithms. This dataset, derived from 11 mouse brain samples imaged using fluorescence micro-optical sectioning tomography, contains carefully selected 852 volume images sized at 192 × 192 × 192 voxels. These images exhibit substantial variations in terms of axon density, image intensity, and signal-to-noise ratios, even within localized regions. Conventional methods often struggle when processing such complex data. To address these challenges, we propose a distance field-supervised segmentation network designed to enhance image signals effectively. Our results demonstrate significantly improved axon detection rates across both state-of-the-art and traditional methodologies. The released dataset and benchmark algorithm provide a data foundation for advancing novel axon reconstruction methods and are valuable for accelerating the reconstruction of long-range axonal projections.

## Introduction

Neuronal reconstruction is regarded as mining quantitative data characterizing neuronal morphology from brain imaging data (Meijering, 2010; Parekh and Ascoli, 2013). This quantitative data can be used to determine the spatial location of neurons, axonal projection pattern, neuronal protrusion connections, and other important information (Svoboda, 2011; Helmstaedter and Mitra, 2012; Lu, 2011), which play important roles in neuronal identification, brain circuit mapping, neural modeling (Zingg et al., 2014; Gao et al., 2022; Foster et al., 2021; Munoz-Castaneda et al., 2021). Neuronal reconstruction, as one of the core contents of brain spatial informatics, has become a bridge from imaging data to the discovery of new laws and knowledge in neuroscience (Choi et al., 2024; Liu et al., 2022).

Recent advancements in molecular labeling and imaging technologies have facilitated single-cell resolution imaging of whole-brain neuronal morphologies, thereby advancing brain structure research to unprecedented levels (Li et al., 2010; Chung and Deisseroth, 2013; Osten and Margrie, 2013; Economo et al., 2016; Cai et al., 2019). Industrial-scale throughput now generates hundreds of terabytes of neuronal morphology data on a daily basis, encompassing tens of thousands of whole-brain neurons. Current whole-brain reconstruction pipelines predominantly rely on semi-automated methods (Winnubst et al., 2019; Wang et al., 2019; Zhou et al., 2021; Gou et al., 2024); however, the speed of reconstruction remains significantly slower than the rates at which data are acquired. This bottleneck arises from the fact that long-range axons of these neurons traverse entire brain volumes, with their structural signals dispersed across hundreds of gigabytes to terabytes of imaging data. As a result, a substantial portion of computational resources during reconstruction is consumed by axon tracing processes (Wang et al., 2019; Gou et al., 2024).

The majority of neuron reconstruction algorithms focus on dendrites reconstruction, which involves two categories: sparse and dense reconstruction methods. Sparse reconstruction methods primarily exploit the local orientation of dendrites to establish point-to-point connections along dendritic skeletons (Brown et al., 2011; Manubens-Gil et al., 2023; Wang et al., 2011; Feng et al., 2015; Xiao and Peng, 2013; Wearne et al., 2005; Radojevic and Meijering, 2017; Athey et al., 2022). Notably, the Diadem (Brown et al., 2011) and BigNeuron (Manubens-Gil et al., 2023) projects, through standardized testing platforms and unified datasets, accelerated the development of advanced algorithms including variational methods (Wang et al., 2011), graph-based approaches (Feng et al., 2015; Xiao and Peng, 2013), and region-growing techniques (Wearne et al., 2005). Dense reconstruction methods, such as NeuroGPS-Tree (Quan et al., 2016), NeuronCyto II (Ong et al., 2016), PAT (Skibbe et al., 2019), G-Cut (Li et al., 2019), and progressive learning-based reconstruction framework (Chen et al., 2021), were subsequently proposed. These methods isolate individual neuronal morphologies from population-level data. Advances in deep learning have significantly enhanced the accuracy of dendrite reconstruction, particularly in identifying neuronal foreground regions (Li et al., 2017; Liu et al., 2022; Chen et al., 2023; Jin et al., 2024; Yao et al., 2024). Specifically, a probabilistic map of the foreground region is obtained by constructing a neural network, and then, this probabilistic map is enlarged and superimposed on the original image to enhance the dendrite image intensity, which improves the reconstruction accuracy of dendrites in neurons.

The integration of deep learning into the dendrite reconstruction framework described above holds promise for extending to axonal reconstruction. However, the complexity of axonal reconstruction significantly exceeds that of dendritic tasks (Yao et al., 2024), presenting specific challenges at two levels: on one hand, axonal extensions can span several orders of magnitude greater than dendrites. This expansive spatial scale increases uncertainties during sample preparation and imaging processes, resulting in considerably broader signal variations. On the other hand, the narrow diameter of axons hinders molecular diffusion for labeling purposes. Certain regions within axons exhibit sparse labeling molecules, leading to substantial weak signals in neuron images where foreground-background contrasts are minimal (Huang et al., 2020). Furthermore, the publicly available BigNeuron dataset contains limited axonal data. This data imbalance further complicates efforts to develop and evaluate algorithms for axonal reconstruction (Manubens-Gil et al., 2023; Chen et al., 2024). Consequently, constructing datasets that incorporate morphological features of axons has become a prerequisite for establishing high-accuracy reconstruction methodologies.

This work presented the axonal dataset and performed detailed morphological annotation. This dataset includes 852 axonal data blocks carefully selected from 11 whole rat brain imaging data collected from fluorescence micro-optical sectioning tomography system (fMOST) (Wang et al., 2021). We subsequently applied a distance field supervised U-Net architecture to process these datasets (Çiçek et al., 2016), thereby establishing baseline results for future methodological comparisons. The dataset demonstrates remarkable feature diversity, encompassing a wide range of signal-to-noise ratios and varying distributions of axonal density. This work provides a robust data foundation for the development of deep learning-driven axonal segmentation algorithms, with the potential to enhance the precision of neuronal 3D reconstruction and achieve significant advancements in large-scale neural circuit mapping efficiency.

## Methods

### Data collection

The dataset was collected using fluorescence micro-optical sectioning tomography (fMOST) imaging technology (Wang et al., 2021). The specific procedure is outlined below: Initially, biological samples underwent dual processing involving AAV viral tracer labeling and resin embedding to achieve precise visualization of target neural circuits at sub-micron resolution. Subsequently, the samples were subjected to micron-scale continuous sectioning and layer-by-layer scanning imaging via the fMOST system. This was combined with chemical sectioning fluorescence tomography to suppress background fluorescence, thereby significantly enhancing the imaging signal-to-noise ratio. Finally, image stitching and motion artifact correction were performed concurrently during data acquisition to generate a comprehensive high-throughput three-dimensional dataset of the mouse brain. In our analysis, a total of 11 mouse brain imaging data are used. In these datasets, axonal projection fields primarily encompassing the Entorhinal Cortex, Perirhinal Cortex, Ectorhinal Cortex, Ventral Auditory Cortex, Visual Cortex, Pontine Reticular Nucleus.

The imaging data for a single mouse brain amounts to approximately 10 TB. From this dataset, we selected some representative data blocks, each of size 192 × 192 × 192. The specific selection process is as follows: First, each mouse brain dataset was converted into the Tdat pyramid data format (Li et al., 2017). Next, the three-dimensional mouse brain was divided into data blocks of size 192 × 192 × 192 using the pyramid data format. Then, grayscale histograms of these data blocks were computed and clustered into 1,000 classes using the K-means method, with each class contributing one data block. In other words, the algorithm initially auto-selected 1,000 data blocks. Finally, from these 1,000 data blocks, we manually selected a subset ranging from 70 to 100 for further analysis.

### Deep learning segmentation network

For neural image segmentation tasks, we implement a 3D U-Net architecture (Çiçek et al., 2016). The encoder features six convolutional blocks with channel dimensions progressively doubling from 16 to 512. The decoder mirrors this structure using six convolutional blocks with 3D transposed convolutions (stride = 2) for spatial upsampling. Residual skip connections, following ResNet structure, progressively fuse multi-scale features from encoder and decoder stages to preserve structural details. Detailed architectural specifications are provided in reference.

Experimental implementation was performed in PyTorch 2.1 on an NVIDIA GeForce GTX 1080 Ti GPU. Training employed the Adam optimizer with initial learning rate 2 × 10^−4^ and batch size of 1. The dataset comprised 852 manually annotated neuronal images displaying diverse signal-to-noise ratios and axonal densities, divided into train (676), validation (85) and test (91) datasets. Network training utilized *L_1_* loss defined as Equation (1):


(1) $$\begin{array}{l} \text{Loss}(y_{p},y_{g})=\\ \begin{array}{l} \frac{1}{N_{\text{total}}}{\left\|y_{p}-y_{g}\right\|}_{1}+\frac{1}{\#(\mathrm{re}g_{1})}{\left\|{\left(y_{p}-y_{g}\right)}_{\mathrm{re}g_{1}}\right\|}_{1}\\ +\frac{1}{\#(\mathrm{re}g_{2})}{\left\|{\left(y_{p}-y_{g}\right)}_{\mathrm{re}g_{2}}\right\|}_{1}+\frac{1}{\#(\mathrm{re}g_{2}^{*})}{\left\|{\left(y_{p}-y_{g}\right)}_{\mathrm{re}g_{2}^{*}}\right\|}_{1} \end{array} \end{array}$$

Here, $y_{p}$ and $y_{g}$ represent the segmentation network output and the ground truth, respectively. $\mathrm{re}g_{1}$ and $\mathrm{re}g_{2}$ are the regions of the ground truth image where the voxel intensity exceeds more 3/255 and103/255, respectively. $\mathrm{re}g_{2}^{*}$ is the region of the segmentation input in which the voxel intensity is more than 103/255. # denotes the total number of voxels in the region. Denote that the operation of subtraction is performed in the *region*.

### Training datasets

**Figure 1:**
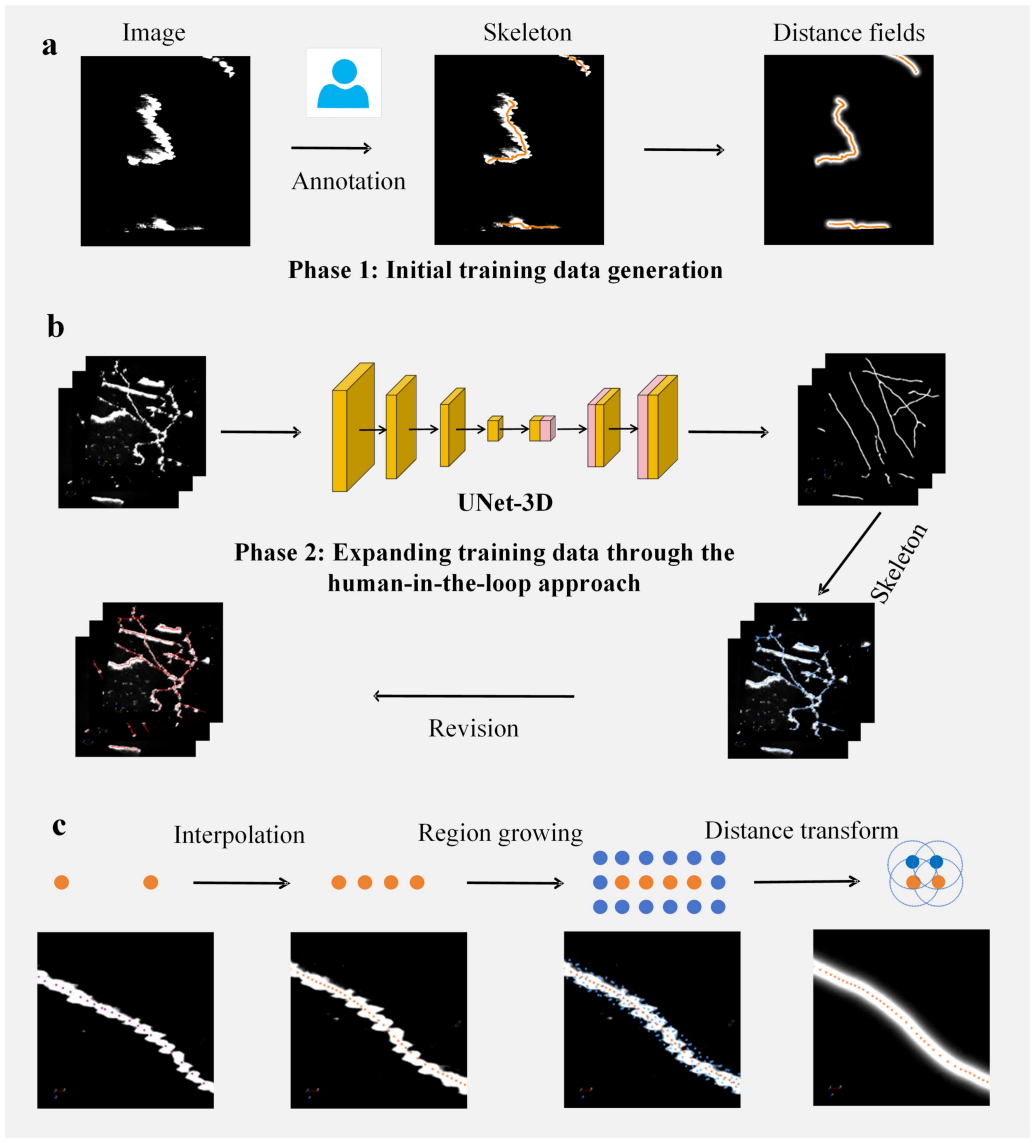
Workflow for constructing the training set. **(a)** Initial training set acquisition. Manual annotation is performed on input images to obtain axonal centerlines (skeletons). These skeletons are subsequently utilized to generate distance fields, thereby forming the initial supervised labels. **(b)** Human-in-the-loop training set augmentation: Step 1 involves training a segmentation network using the initial training set and applying it to predict new data; Step 2 entails automatic extraction of skeletons from the predicted data; Step 3 consists of manual revision of the extracted skeletons to produce network label maps, which are then combined with the initial set to create an augmented training set; Step 4 retrains the network and further expands the data through repeated execution of Steps 1–3. **(c)** Supervised label generation protocol. This process comprises three stages. Skeleton resampling: interpolation ensures that adjacent skeleton points are spaced ≤1 voxel apart; Region expansion: spherical regions with a radius of 3 voxels centered at each skeleton point are merged to form expanded domains; Distance field generation. The characteristic of this distance field is that the closer a voxel’s position is to the axonal centerline, the greater its value will be. The white regions in the first three figures represent axonal signals.

In the process of constructing the training set, we employed a human-in-the-loop approach to perform annotations. The entire workflow for neuron image annotation is illustrated in Figure 1. This process consists of two stages: the first stage involves obtaining an initial segmentation network (Figure 1a); the second stage utilizes this initial network to make predictions on images, thereby augmenting the training set, enhancing the accuracy of the network, and accelerating the annotation process (Figure 1b).

In the first phase of constructing the training set, we utilized the GTree tool (Zhou et al., 2021) for semi-automated analysis of 50 data blocks to obtain their skeletal structures. In cases where the reconstructed skeletons were incorrect, manual revisions were made. All annotators were directly supervised by experienced neuroanatomists, with regular cross-validation conducted among annotators to ensure consistency. Once the skeletons were acquired, interpolation was performed on critical skeletal points to ensure that the distance between adjacent skeletal points did not exceed one voxel unit. Subsequently, an expansion was conducted around each skeletal point to derive expanded regions. These expanded regions and skeletons were then employed to generate label maps for a supervised segmentation network. Specifically, a given axonal skeleton is denoted as $S=\{s_{0},s_{1},\cdots ,s_{n}\}$ , where $s_{i}$ represents a three-dimensional vector indicating the position of the i-th skeletal point. The expanded region is calculated according to Equation (2):


(2) $$S_{\mathrm{Reg}}=\{v|\min\{\|v-s_{i}\|,i=1,2,\cdots ,n\}<3\}$$

The variable *v* represents the coordinates of the voxels within the image. The aforementioned indicates that the expanded region is formed by the union of multiple spherical areas, each centered around a skeleton point and encompassing an internal radius of 3 units. Subsequently, we calculate the distance between voxel *p* in the expanded region and the central axis using Equation (3):


(3) $$\text{dist}(p,s)={\left\|p-s_{\left(p\right)}\right\|}_{2}\sin(\text{acos}(<\vec{s_{\left(p\right)}p},\vec{s_{\left(p\right)}s_{(p)+1}}>))$$

The point represents a point in the skeleton that is closest to point *p*. In this expression, the direction vector has a unit length of 1. The meaning of the expression is to calculate the perpendicular distance from the vector (starting at $s_{\left(p\right)}$ and ending at *p*) to the direction (starting at and pointing toward $s_{(p)+1}$ ). For each point in the expanded area, we compute the distance from that point to the centerline using the expression (3). The calculation of the distance field follow Equation (4):


(4) $$\text{disf}(p,s)=e^{-\;\frac{1}{2\sigma^{2}}\text{dist}(p,s)}$$

Figure 1c illustrates the process of generating a distance field based on the axonal skeleton. We employ this distance field to supervise the segmentation network. The output of the network resembles a distance field, where the values are maximized at the center of the axonal skeleton and decrease as one moves away from it. This type of supervisory approach has been widely applied in biomedical image analysis (Stringer et al., 2021).

After obtaining the initial segmentation model, we process the distance field information output by the network. Regions of the image that exceed a certain threshold are classified as foreground areas. These foreground voxels, when considered in spatial positions, form a point set. We employ the PointTree method (Cai et al., 2024) to handle this point set, which allows for the automatic and accurate generation of axonal skeletons, referred to as predicted skeletons. The predicted skeletons are then revised using GTree software (Zhou et al., 2021); these modified skeletons produce distance fields. This approach expands our training dataset, and with this augmented training set, we retrain the network to improve its accuracy and generalization capabilities. We update our training network two times to obtain annotations for our training image, specifically, the axonal skeletons.

### Tracing algorithms description

The crucial step in neuron reconstruction is to extract the neuronal process skeletons from neuronal images and establish connections between these skeletons. The tracing algorithm serves as the ways to achieve this critical step. We selected several typical tracing algorithms including neuTube (Feng et al., 2015), Open-snake (Wang et al., 2011) and PHDF (Radojevic and Meijering, 2017), as benchmark algorithms to test the data we submitted. neuTube is recognized as a highly competitive and widely adopted method. Open-snake is a method recommended by the DIADEM neuron reconstruction project. PHDF is a probabilistic model-based approach demonstrating competitiveness in skeleton extraction. Given the complexity and diversity of the initial images, the likelihood that optimal parameter settings will significantly enhance the performance of tracing algorithms is relatively low. When the segmentation network identifies neuronal process regions from these initial images and increases the image intensity of these regions, it implies a significant improvement in the image’s signal-to-noise ratio. Under such circumstances, there exists a very broad range of parameter settings that enable the algorithm to achieve stable tracing results (Liu et al., 2021).

### Evaluation metrics

Considering that axonal segmentation is primarily applied to enhance the signal of the initial images, we evaluate the effectiveness of axonal segmentation using the following method. The neural image annotations contain information about the centerline of the axons, specifically the connections between skeleton points. This information is stored in SWC files (Ascoli et al., 2001). We assess the effectiveness of network segmentation by examining the differences between real skeletons and those generated by our network. The rationale behind this evaluation method is also reflected in how we construct our training set; supervised segmentation networks utilize neural image labels derived from axonal skeletons. As long as there is a strong consistency between predicted skeletons and manually annotated ones, their label maps will also maintain a good level of consistency.

The skeleton generated through network induction is denoted as *P*, while the manually annotated skeleton is referred to as *G*. The differences between *P* and *G* are evaluated using metrics such as recall, precision, and F1 score. To compute these metrics, we first define a function as Equation (5):


(5) $$f(p,G,\text{Thre})=\{\begin{array}{c} 1,\min\{{\left\|p-g_{p}\right\|}_{2},g_{p}\in G\}<\text{Thre}\\ 0,\;\text{otherwise}\;\; \end{array}$$

Here, the distance between *p* and *g_p_* is measured by 2-norm squared. *Thre* representing the threshold set to 3. If the distance between a point *p* in the skeleton point set *P* and its nearest corresponding point *g_p_* in the manually labeled skeleton G is less than *Thre*, then the function value is assigned as 1; otherwise, it is assigned as 0. Based on this definition of the function, we calculate recall, precision, and F1 score using Equations (6–8):


(6) $$\text{Recall}(P,G)=\frac{\sum_{g_{j}\in G}f(g_{j},P,\text{thre})}{N_{G}}$$

(7) $$\text{Precision}(P,G,t)=\frac{\sum_{p_{i}\in P}f(p_{i},G,\text{Thre})}{N_{P}}$$

(8) $$F1=2\frac{\text{Recall}\times \text{Precision}}{\text{Recall}+\text{Precision}}$$

In the calculation of the recall rate formula, *N_G_* represents the number of skeleton points identified manually, while *N_P_* denotes the number of skeleton points derived from the segmentation network’s output.

To evaluate the reconstruction performance of axonal topological structures, we select the AJI (Aggregated Jaccard Index; Kumar et al., 2017) metric as the evaluation criterion. Specifically, for manually labeled axonal skeletons, we generate the regions where axons are located in the image based on Formula 2. When skeletons are interconnected, the regions generated from these skeletons share the same identifier. By following this method, we obtain the manually labeled instance segmentation results of axons, denoted as ${\mathrm{Gc}}_{i}$ . Correspondingly, after reconstructing the axonal skeletons in neural images using the algorithm, we can generate the algorithm-derived instance segmentation results, denoted as ${\mathrm{Pc}}_{j}$ . Finally, the AJI value is calculated using Equation (9):


(9) $$\mathrm{AJI}=\frac{\sum_{(i,j)\in \text{matched}\;\;\text{pairs}}|{\mathrm{Gc}}_{i}\cap {\mathrm{Pc}}_{j}|}{\sum_{(i,j)\in \text{matched}\;\;\text{pairs}}|{\mathrm{Gc}}_{i}\cup {\mathrm{Pc}}_{j}|+\sum_{k\in \text{unmatched}\;\;}|{\mathrm{Gc}}_{k}|+\sum_{l\in \text{unmatched}}|{\mathrm{Pc}}_{l}|}$$

Here ∣∣ represents the total number of voxels in the given region. If (i,j)∈mathed paires , the region ${\mathrm{Pc}}_{j}$ has the largest Jaccard Index with ${\mathrm{Gc}}_{i}$ .

## Results

### Data diversity estimation

**Figure 2:**
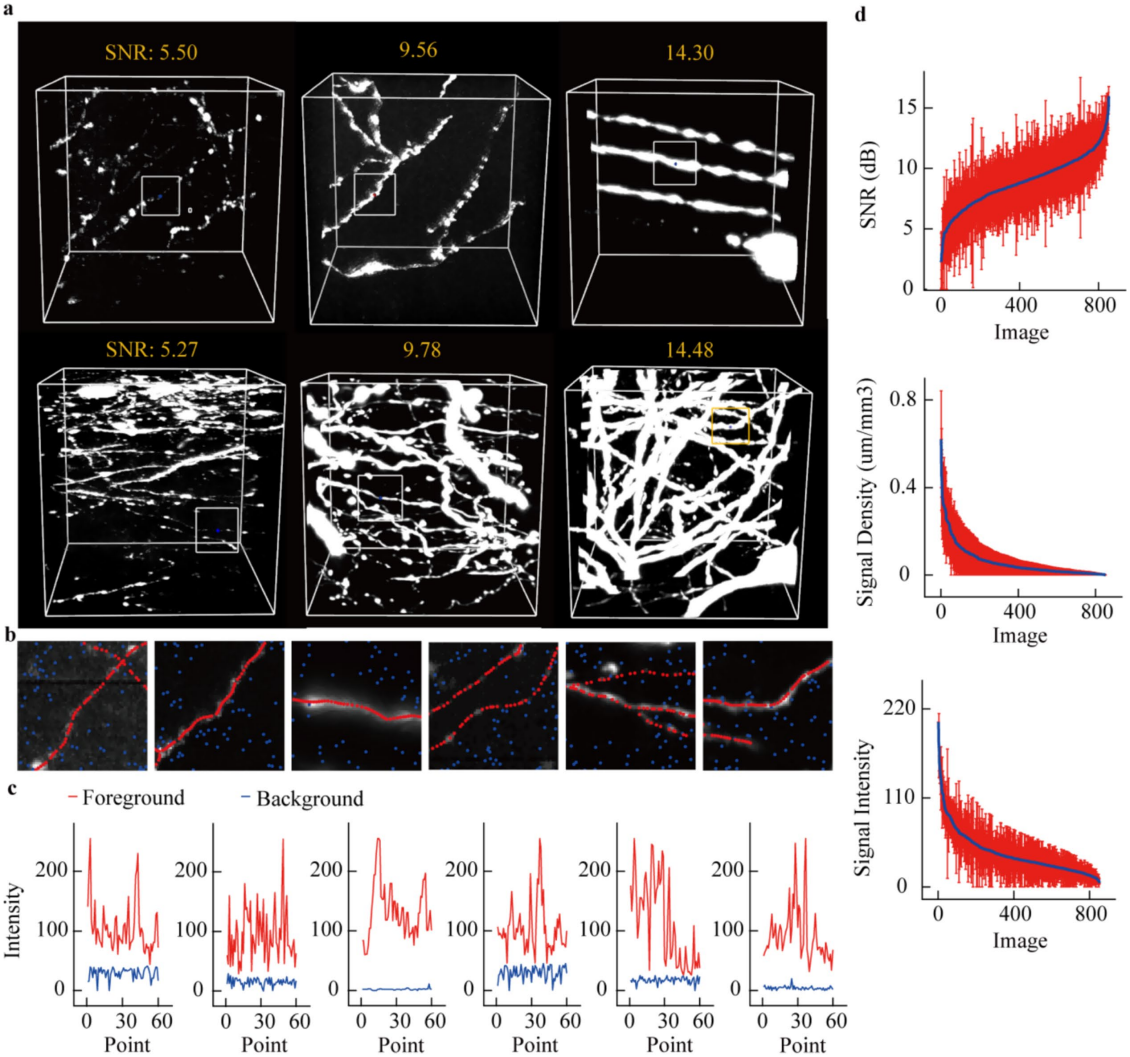
Quantitative evaluation of neuronal axon images. **(a)** Typical display of several neuronal image data blocks. These data blocks include axons with different signal-to-noise ratios and different distribution densities. **(b)** More refined display of axonal signals. Select a local area from each data block in **(a)** to show the intensity of the axonal image and the background nearby. The red dots represent the skeleton points of the axon, and the blue dots represent the background. **(c)** Display the image intensity of the axonal skeleton points and the background point intensity for each sub-region. **(d)** Utilize signal-to-noise ratio, axon distribution density, and image intensity to characterize axonal image features. For each image block, further divide it into 27 sub-blocks, and use the average and variance of the feature metrics of these sub-blocks to characterize the image block. The average signal-to-noise ratio of the image is ranked in ascending order, and the red vertical line represents the standard deviation. The signal density and image intensity are arranged in descending order. The x-axes of the three subplots all represent data block identifiers, but the meanings of these identifiers differ. The x-axis identifier i denotes the data block with the i-th largest average signal-to-noise ratio (Top), i-th smallest axon distribution density (Middle) and i-th smallest image intensity (Bottom).

First, we assessed the diversity of this datasets based on signal-to-noise ratio (SNR), axon distribution density, and signal intensity. Figure 2a displays six groups of images organized in two rows and three columns. From left to right, there is a progressive increase in the SNR of the images; axons in the first row demonstrate sparse distribution, whereas those in the second row exhibit dense distribution. To emphasize the contrast between signals and background within these images, we selected specific local regions for analysis (Figure 2b). Subsequently, we present the intensity values for skeletal points as well as background regions (Figure 2c). The following characteristics can be summarized from Figure 2c: firstly, even in areas with high SNR, weak signals persist, exhibiting minimal differences from the background. Secondly, signal intensity shows considerable fluctuations even within a relatively narrow data range. Finally, despite notable variations in background intensity across different images, overall fluctuations in background intensity remain limited.

To further characterize these data precisely, each image volume (192 × 192 × 192) was partitioned into smaller blocks of 64 × 64 × 64 voxels. For each block, we calculated the signal-to-noise ratio (SNR), axon distribution density, and axon signal intensity. These values were subsequently averaged, and their variances were computed. Through this process, we derived the SNR, axon density, and signal intensity metrics for the entire datasets. As illustrated in the Figure 2d, SNR values range from 2.5 to 15, with substantial fluctuations observed in sub-cube data (standard deviation ≈3.5). Quantitative analysis of axon distribution reveals a highly dense subset of data; however, the majority remains relatively sparse. Similarly, signal intensity across different datasets demonstrates a high proportion of weak signals. Notably, the large standard deviation in signal intensity partially arises from regions containing both extremely strong and weak signals simultaneously, as exemplified in Figure 2c. These results collectively indicate that even within a localized spatial range, the features of axon images exhibit remarkable variability.

### Benchmark results of network segmentation

**Figure 3:**
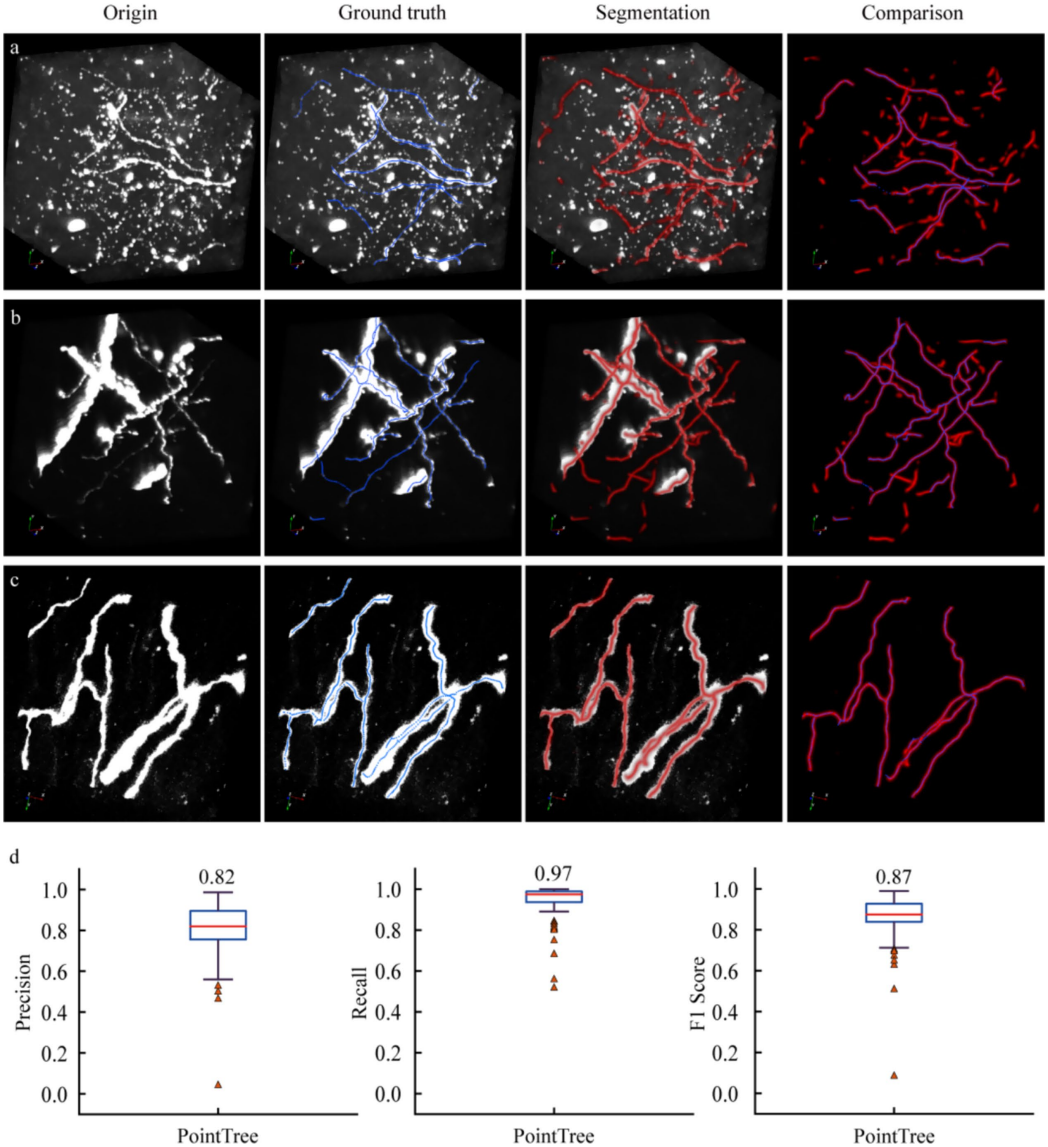
Performance of segmentation networks in axon identification. **(a)** Axon identification in complex scenes. There are a large number of interference signals in neural images, and some interference structures are easily misidentified as axons. **(b)** Identify scenes with high accuracy. The vast majority of axons were correctly identified, with only a very small number of false identifications. **(c)** Recognize scenes with high accuracy. All axons were accurately identified, with no false positive results. **(d)** Quantify the results of axon recognition derived from the segmentation network. The distance field output by the segmentation network is processed using PointTree to directly generate a skeleton. The accuracy, recall, and F1-score are obtained by comparing the algorithm-generated skeleton with the manually revised skeleton.

We employed the 3D U-Net model to segment these data and evaluated the segmentation results by comparing the skeleton generated through network prediction with the manually annotated skeleton. Three representative datasets were demonstrated, which included: numerous non-axonal structures with diverse morphological features. For non-axonal data, their diverse manifestations (round or strip-shaped) posed challenges. The segmentation network tended to misclassify strip-shaped structures as axons, thereby reducing segmentation accuracy (first row of Figure 3a). For the second dataset (second row of Figure 3b), weak axonal signals could still be accurately segmented, meanwhile strong axonal signals with large-radius cross-section achieved satisfactory results. The third dataset (third row of Figure 3c) demonstrated precise segmentation of axons. These results indicated that the network achieved high recall but relatively low precision, as some non-axonal signals were erroneously identified as axons. The segmentation network exhibited robust performance in capturing various axonal morphologies but required optimization to reduce false positives from non-axonal structures.

Further quantitative analysis was conducted on the segmentation results of 90 image blocks. We calculated the recall, precision, and F1-score for these data. The results showed an average recall of 0.97, an average precision of 0.82, and an F1-score of 0.87. The relatively low precision indicated that the segmentation algorithm misclassified some non-axonal signals as axons, while the high recall demonstrated its capability to accurately identify weak axonal signals. Notably, a small subset of data exhibited low recall and precision values. This was primarily attributed to these images containing extremely sparse and weak axonal signals. Overall, the segmentation network achieved satisfactory accuracy in identifying axonal structures within neural images, balancing effective detection of weak signals with moderate precision limitations in complex backgrounds.

### The segmentation results applied in axonal reconstruction

**Figure 4:**
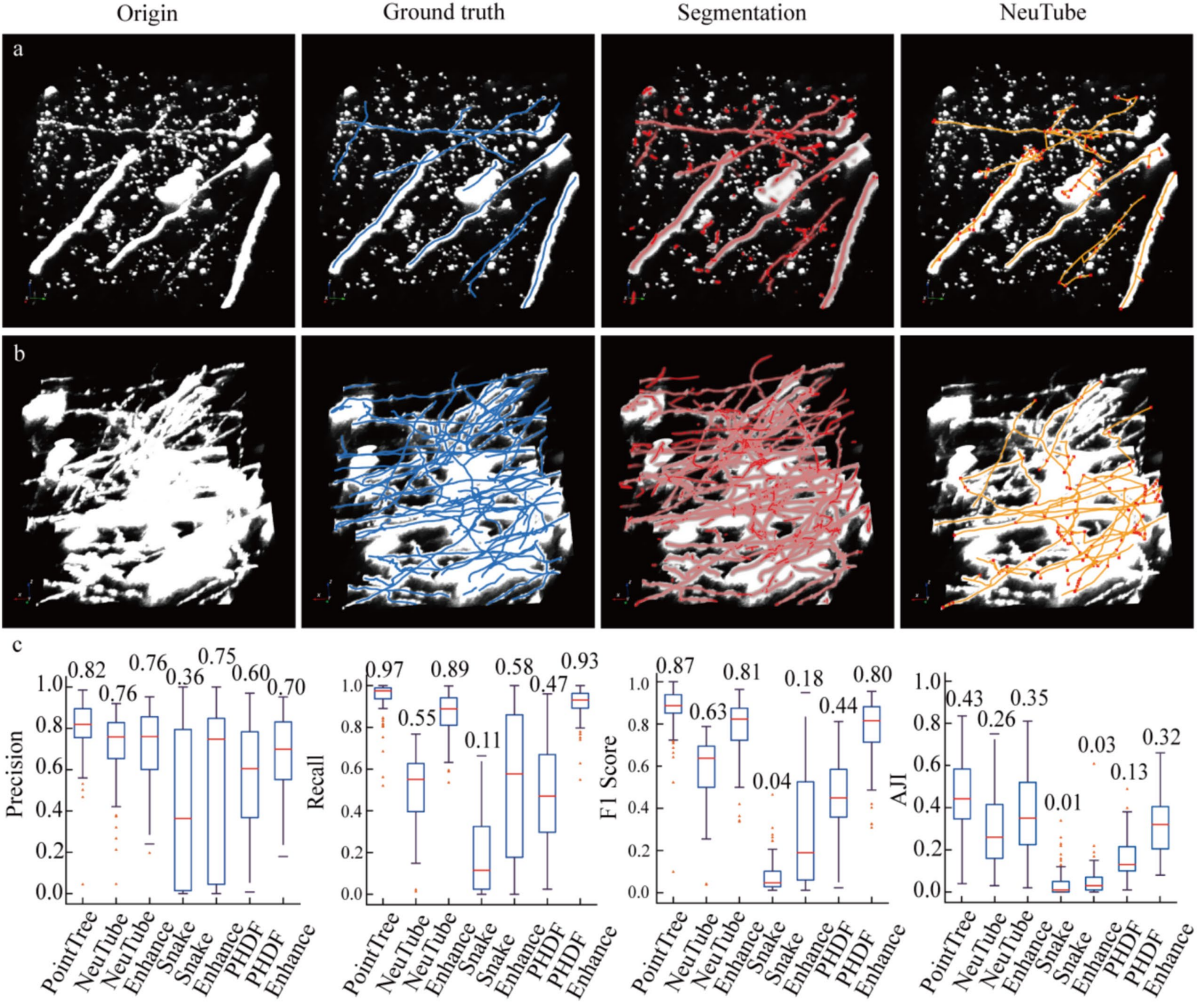
Enhancing image signals using a segmentation network to improve the accuracy of axon reconstruction. **(a)** Data display of sparsely distributed axons and their corresponding automatic reconstruction results. Among the presented data, only the reconstruction results of the NeuTube algorithm on the initial images are shown. **(b)** Data display of relatively densely distributed axons and their automatic reconstruction outcomes. In the original images, some axons appear to have extremely large diameters due to display limitations, which is done to highlight axons with weak signals (i.e., axonal regions with low image intensity values). The boundary image intensity values of some strongly signaled axons can be significantly higher than those of weakly signaled axons. **(c)** Comparison of reconstruction results between image-enhanced and original images. The PointTree method directly extracts the axon skeleton from the segmented image without involving reconstruction from the raw data. The evaluation results of the reconstructions are presented using box plots, which show the median values, as well as the upper and lower quartiles.

We demonstrate that segmenting neuronal axons significantly improves the accuracy of their reconstruction. To validate this, two groups of experiments were conducted: one where algorithms reconstructed axons from original data, and another where reconstruction was performed on enhanced data. The data enhancement method involved scaling the network-predicted segmentation map by 255 times and overlaying it onto the original image. For automated tracing algorithms, we employed several typical methods, including neuTube, Snake, and PHDF, using their own parameter settings across all tests without fine-tuning. Additionally, we present reconstruction results from the PointTree algorithm, which directly processes network-predicted segmentation maps to derive axonal centerlines. Figures 4a,b illustrates three datasets: original data, manually labeled skeletons (ground truth), network-predicted distance fields, and automated tracing results from original images. Visual analysis reveals that network-segmented distance fields enhance signal intensity around axonal centerlines, creating clearer contrasts between centerline regions and axon boundaries/background noise. This facilitates more effective extraction of axonal centerlines from neuronal images.

We evaluated the performance of automated algorithms in reconstructing axons from both original and enhanced images (Figure 4c). In original images, the highest recall achieved by the three automated reconstruction algorithms was 0.58, indicating substantial undetectable axons remained. A critical factor contributing to low recall was the requirement for parameter optimization in these algorithms, as fixed parameters could not adapt to image sets containing complex, variable scenarios. Precision in original images showed significant improvement over recall, reaching 0.76. This suggests that in neuronal axon image, the majority of identified axons were correctly detected. When processing enhanced images, these algorithms demonstrated drastically improved recall, with maximum values reaching 0.93, a nearly 0.3 increase. This indicates augmented images not only enhanced weak signals but also reduced image complexity, enabling better algorithm adaptation to enhanced data under fixed parameters. However, precision improvement was less pronounced, potentially due to other signal interference or boundary artifacts. The substantial recall improvement elevated F1-scores from 0.63 to 0.81. Notably, PointTree achieved the highest F1-score of 0.87 by directly processing network-predicted distance fields. We also conducted a quantitative analysis of the results of axon topological structure reconstruction using AJI. Reconstructing axon topological structures not only requires identifying the skeletons of axons but also further establishing the connection relationships between the identified skeletons. The results shown in the last sub-figure of Figure 4c, the enhanced images can still improve the performance of axon topological structure reconstruction.

### The segmentation model applied in cross-modality reconstruction

We evaluated the generalization performance of the model trained using the submitted dataset. For the test data, we selected 10 light-sheet imaging acquisition image stacks, each with size of 192 × 192 × 192 (Cai et al., 2019). The segmentation model was trained by fMOST data, and this model served as our benchmark segmentation model, applied in Figures 3, 4. These 10 datasets were randomly selected. Within the image regions, there were areas with densely distributed axons, as well as situations where the intensity of axon images showed minimal contrast with the surrounding background. We employed the PointTree method to extract skeletons from the segmented images and establish the skeleton connectivity.

**Figure 5:**
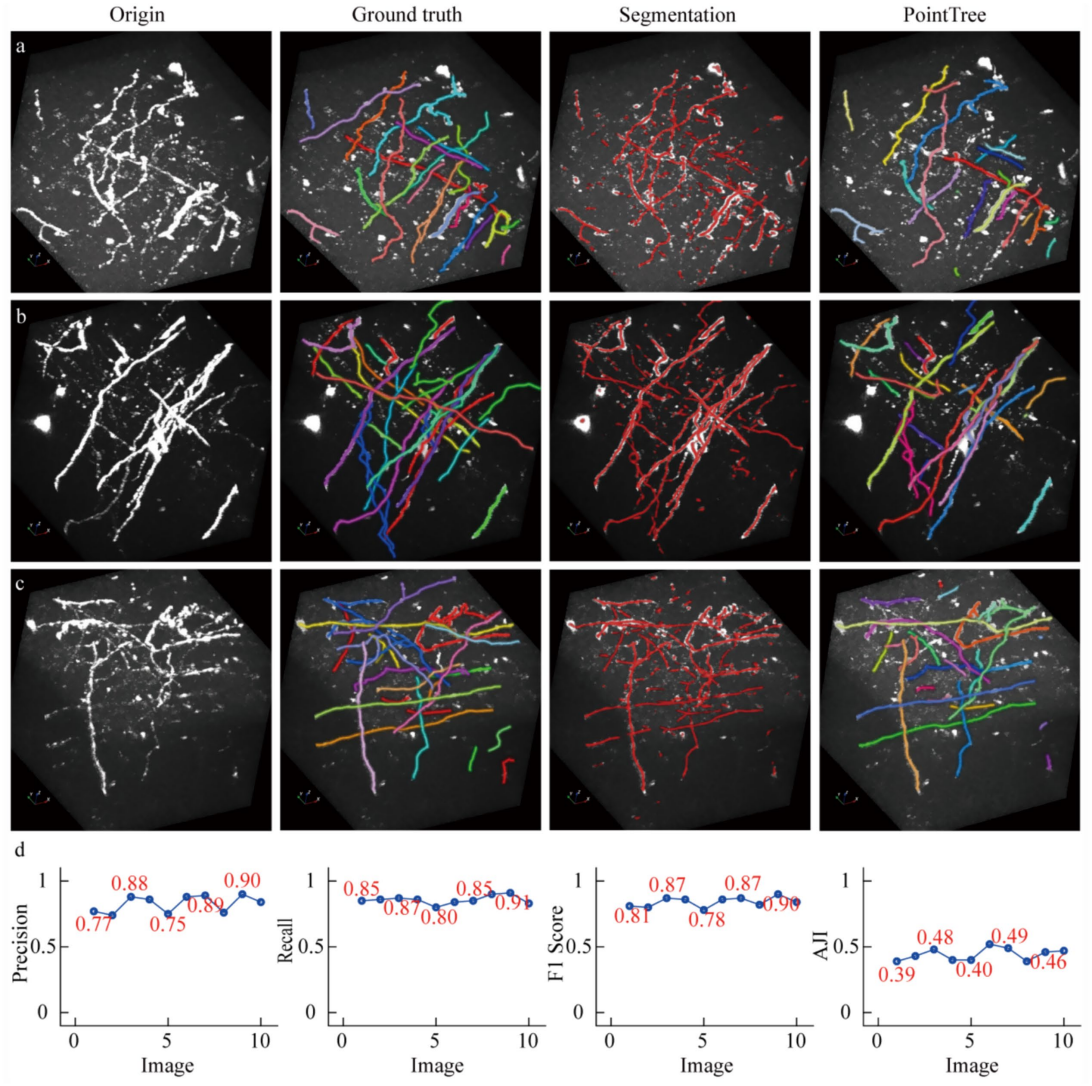
The reconstruction results of light-sheet imaging data using a model trained with fMOST data. **(a–c)** The neural image data acquired by the light-sheet imaging system, along with manually annotated reconstruction results, automatically extracted skeletons, and topological structure reconstruction consistent of the ground truth. **(d)** The quantitative analysis of the reconstruction results, which is based on 10 data blocks. Among them, the first three subplots are used to quantify the recognition rate of axons, a process that does not involve the evaluation of connectivity; the last subplot is employed to assess the reconstructed connectivity.

We presented relevant data, manual annotation results, as well as the automatic skeleton extraction and reconstruction of axons connectivity (Figures 5a–c). Compared with the manual annotation results, the segmentation network was able to identify most axons. However, the network had some shortcomings: on the one hand, some non-neurite signals with strong intensity were misidentified, which led to a decrease in the accuracy of neurite recognition; on the other hand, axon signals with low image intensity were not recognized, resulting in a reduction in the recall rate of axon recognition. We further conducted a quantitative analysis of the reconstruction results of axons. Regarding axons recognition performance, we evaluated these 10 datasets, and the F1-score ranged from 0.78 to 0.9, indicating that the segmentation network could recognize most axons in the light-sheet imaging data. In terms of establishing axons connectivity, that is, topological structure reconstruction, the AJI values ranged from 0.39 to 0.51(Figure 5d). These reconstruction performance is generally at the same level as the results shown in Figure 4, which suggests that our submitted datasets collected by the fMOST system are helpful for the axon reconstruction from the images collected with light-sheet imaging system.

## Discussion and conclusion

Here we present an axon dataset generated by the fMOST system, accompanied by corresponding annotations. Despite being acquired through the fMOST system, this dataset demonstrates remarkable diversity and complexity in its data characteristics. Notably, axon signal intensities can exhibit drastic variations within minimal spatial extents. Furthermore, we demonstrate the recognition capabilities of deep learning networks on these neural image data, showing that recognition-enhanced neural image significantly improves axon reconstruction accuracy. Our dataset and methodology provide a benchmark for subsequent algorithmic development, holding potential for achieving robust and precise axon identification at the whole-brain scale.

When assessing axonal reconstruction, greater emphasis should be placed on the capability to detect weak signals at present. In practice, large-scale automatic reconstruction of axons is an exceedingly difficult task, achievable only under ideal signal-to-noise ratio conditions. Under normal circumstances, manual revisions are required to refine the reconstruction outcomes. Before revision, it is crucial to identify the central axis of each neuronal axon. If a reconstructed axon is discovered to be connected to other axons, this connection is often severed which facilitates manual revision (Winnubst et al., 2019; Gou et al., 2024). The revision process mainly includes establishing connections between different axons and extracting the central skeletons of those axons that the algorithm failed to identify. During manual operations, manually establishing these connections still turns out to be highly efficient. However, the most time-intensive task continues to be extracting axon skeletons that can be undetected by algorithm under conditions of weak signals. The submitted datasets should be beneficial for detecting weak signals in axons.

The data we submitted are derived from a single system, without considering the collection of other types of data. This is partly determined by the characteristics of the data we provided. Firstly, the collected images primarily consist of axons. Secondly, there are numerous signals that interfere with axonal structures. Lastly, some axons not only have high image intensity themselves but also show a significant increase in image intensity near their boundaries. These data characteristics differ markedly from those obtained through other two-photon imaging methods and also present clear distinctions when compared to light-sheet imaging data. On another note, much of the current large-scale precise reconstruction results for axonal projections originate from fMOST imaging technique. This is closely related to one of our key objectives in submitting this data, namely, to enhance the accuracy of large-scale axonal reconstructions.

## Data Availability

All datasets are available in https://zenodo.org/records/15372438. Further inquiries can be directed to the corresponding author.
